# The large, diverse, and robust arsenal of *Ralstonia solanacearum* type III effectors and their in planta functions

**DOI:** 10.1111/mpp.12977

**Published:** 2020-08-08

**Authors:** David Landry, Manuel González‐Fuente, Laurent Deslandes, Nemo Peeters

**Affiliations:** ^1^ Laboratoire des Interactions Plantes Micro‐organismes (LIPM) INRAE, CNRS, Université de Toulouse Castanet‐Tolosan France

**Keywords:** effectome, immunity, *Ralstonia solanacearum*, susceptibility, targets, type III effectors, virulence

## Abstract

The type III secretion system with its delivered type III effectors (T3Es) is one of the main virulence determinants of *Ralstonia solanacearum*, a worldwide devastating plant pathogenic bacterium affecting many crop species. The pan‐effectome of the *R. solanacearum* species complex has been exhaustively identified and is composed of more than 100 different T3Es. Among the reported strains, their content ranges from 45 to 76 T3Es. This considerably large and varied effectome could be considered one of the factors contributing to the wide host range of *R. solanacearum*. In order to understand how *R. solanacearum* uses its T3Es to subvert the host cellular processes, many functional studies have been conducted over the last three decades. It has been shown that *R. solanacearum* effectors, as those from other plant pathogens, can suppress plant defence mechanisms, modulate the host metabolism, or avoid bacterial recognition through a wide variety of molecular mechanisms. *R. solanacearum* T3Es can also be perceived by the plant and trigger immune responses. To date, the molecular mechanisms employed by *R. solanacearum* T3Es to modulate these host processes have been described for a growing number of T3Es, although they remain unknown for the majority of them. In this microreview, we summarize and discuss the current knowledge on the characterized *R. solanacearum* species complex T3Es.

## Graphical abstract

This review compiles all the current functional knowledge on the large and diverse repertoire of type III effectors from bacteria belonging to the *Ralstonia solanacearum* species complex.

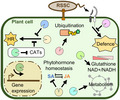

## 1 INTRODUCTION

Bacteria from the *Ralstonia solanacearum* species complex (RSSC) are soilborne plant pathogens responsible for bacterial wilt on more than 250 species, moko and blood diseases of banana, brown rot of potato, and Sumatra disease on clove trees (Peeters *et al*., 2013a). Due to its aggressiveness, broad host range, widespread geographical distribution, and long‐lasting persistence on the soil, *Ralstonia* ranks among the most devastating plant pathogenic bacteria (Mansfield *et al*., 2012). For a successful infection, RSSC bacteria rely on different virulence determinants, including the production of exopolysaccharides and phytohormones, secretion of cell wall‐degrading enzymes, detoxification, and nutrient‐scavenging systems and motility (Genin and Denny, 2012). However, the main virulence determinant of RSSC bacteria is the type III secretion system (T3SS), a “molecular syringe” that allows the translocation of several type III effector proteins (T3Es) directly into the host cell (Coll and Valls, 2013). These T3Es, referred to as Ralstonia injected proteins (Rips), are able to subvert the defences and modify the metabolism of the host to promote virulence.

## 2 THE RSSC TYPE III EFFECTOME, A LARGE AND VARIED ARSENAL

Since the first RSSC *T3E* genes were cloned in the 1990s (Carney and Denny, 1990; Arlat *et al*., 1994; Guéneron *et al*., 2000), different approaches have been conducted to systematically identify at the genome scale the full T3E repertoire of several RSSC strains. Two main strategies were undertaken: (a) sequence‐based approaches, searching for sequence homology with previously described effector genes and/or for the presence of certain 25‐nucleotide *cis* elements in their promoters, the *hrp*_II_ box or the plant‐inducible promoter (PIP) box motifs (Salanoubat *et al*., 2002; Cunnac *et al*., 2004a; Gabriel *et al*., 2006; Peeters *et al*., 2013b; Sabbagh *et al*., 2019), and (b) regulation‐based strategies, exploiting that *T3E* gene expression is controlled by HrpB, an AraC family member of transcriptional regulators (Genin *et al*., 1992; Cunnac *et al*., 2004a). Regulation‐based strategies include gene expression studies (Cunnac *et al*., 2004b; Occhialini *et al*., 2005) and genetic screens using random transposon‐insertion mutagenesis (Mukaihara *et al*., 2004). Verification of the T3SS‐dependency of the secretion or translocation is typically required to confirm the bona fide T3E status of in silico predicted or candidate T3Es (Lonjon *et al*., 2018). Most translocation analyses exploit the adenylate cyclase (Cya) reporter system (Cunnac *et al*., 2004b; Mukaihara and Tamura, 2009; Mukaihara *et al*., 2010). T3SS‐dependent secretion analyses compare the secreted proteins, detected by immunoblotting or mass spectrometry, of wild‐type compared to *hrp* mutant strains (Tamura *et al*., 2005; Solé *et al*., 2012; Lonjon *et al*., 2016; Sabbagh *et al*., 2019).

A recent genomic study on 140 RSSC strains identified the pan‐effectome of the species complex, consisting of 102 *T3E* and 16 hypothetical *T3E* genes (Sabbagh *et al*., 2019). RSSC strains carry on average 64 *T3E* genes (minimum 45 in *R. syzygii* subsp. *syzygii* strain R24 and maximum 76 in *R. pseudosolanacearum* strain Rs‐10‐244). This contrasts with other plant pathogenic bacteria such as *Pseudomonas syringae* and *Xanthomonas campestris*, with an average of 31 (min. 3, max. 53) and 23 (min. 12, max. 28) *T3E* genes, respectively (Roux *et al*., 2015; Dillon *et al*., 2019). The existence of several paralog families, such as the RipG (former GALA), RipS (SKWP), RipA (AWR), RipH (HLK), or RipP (PopP) families, can be considered as a remarkable feature of the RSSC. Not a single known RSSC strain does not carry multiple copies of these paralog T3E families. This contributes to the large size of the RSSC pan‐effectome. The T3E repertoires of different RSSC strains are quite diverse, with only 16 core T3Es (i.e., T3Es present in at least 95% of sequenced strains), which represents 13.6% of the RSSC pan‐effectome (Sabbagh *et al*., 2019). This core‐effectome is larger than in *P. syringae* (four core T3Es, 5.7% of its pan‐effectome) or *X. campestris* (three core T3Es, 8.6% of its pan‐effectome) (Roux *et al*., 2015; Dillon *et al*., 2019). Several studies have tried to connect the T3E diversity to the host specificity of RSSC strains (Ailloud *et al*., 2015; Cho *et al*., 2019; Sabbagh *et al*., 2019). Although some host specificity determinants could be identified, the power of such studies has usually been largely limited by the lack of exhaustive strain host range empirical data.

## 3 MANY T3ES, BUT FOR WHAT PURPOSE?

**FIGURE 1:**
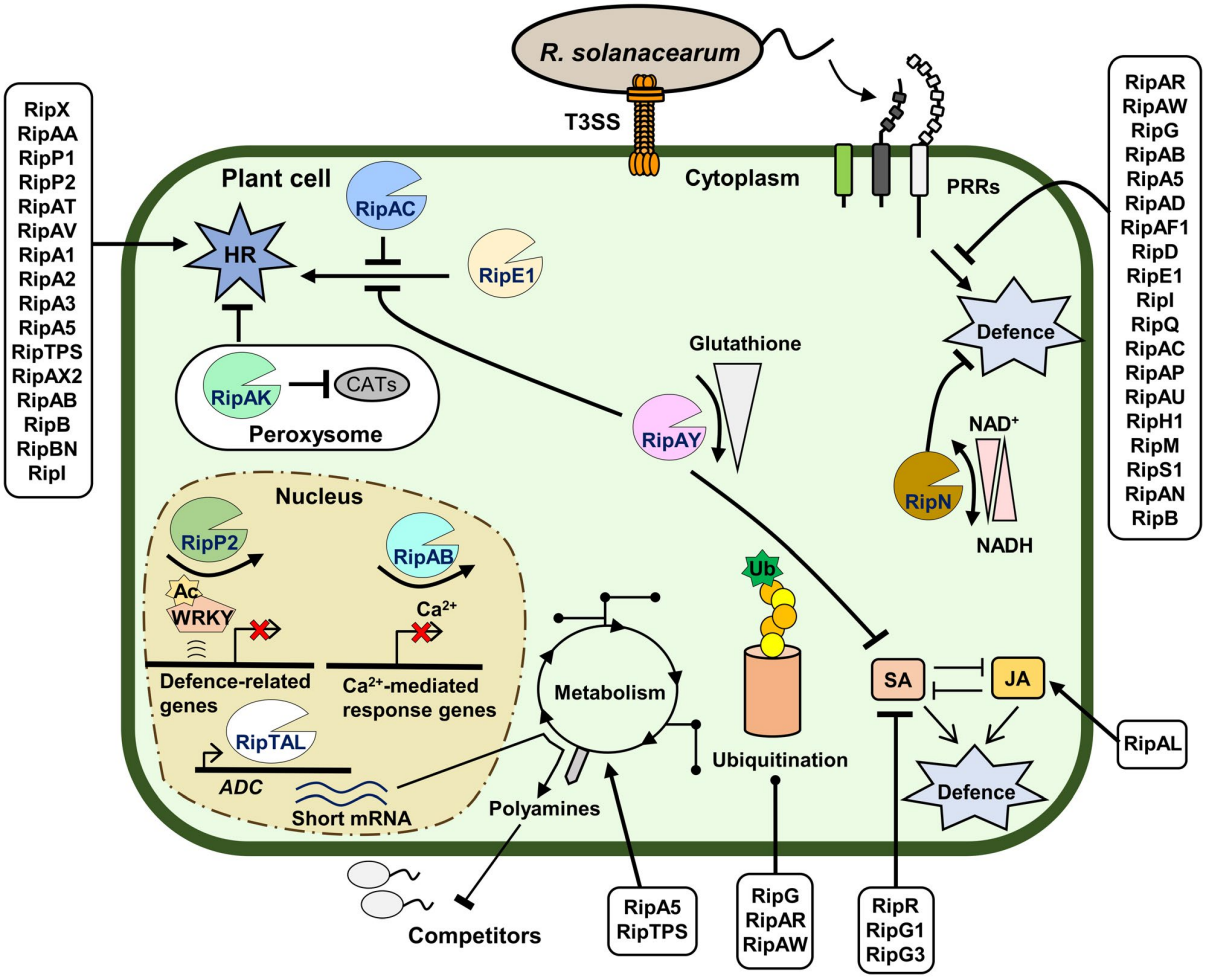
*R* *alstonia solanacearum* species complex (RSSC) bacteria deploy an arsenal of type III effectors (T3Es) to alter the plant metabolism and interfere with plant immune responses. During the infection process, conserved bacterial molecules are recognized by plant pattern recognition receptors (PRRs) at the surface of the host cell. They activate basal defence responses to prevent pathogen proliferation. However, RSSC bacteria translocate T3Es into the plant cell to subvert the plant defences and accommodate the bacterial needs. T3Es act on different host pathways. RipAY and RipN alter the glutathione level and NADH/NAD^+^ ratio, respectively. RipAY, RipR, RipAL, RipG1, and RipG3 target the hormone synthesis and signalling level. Different RipG family members, RipAR and RipAW, interfere with ubiquitination processes. The metabolism is also manipulated by RSSC T3Es. RipA5, RipTPS, and RipTAL are able to modulate certain metabolic pathways. RipTAL binds to the plant DNA, activating the expression of shorter and more efficiently translated transcripts of *arginine decarboxylase* (*ADC*) genes, key enzymes in the biosynthesis of polyamines. This boost in the polyamine level could prevent the proliferation of *Ralstonia* niche competitors. RipP2 relies on its acetyltransferase activity to acetylate defensive WRKY transcription factors, inhibiting their DNA‐binding activities and preventing subsequent expression of defence‐related genes. The nuclear T3E RipAB inhibits the expression of Ca^2+^‐related defence genes. In addition to these functionally characterized RSSC T3Es, other effectors involved in dampening of basal defence through as yet unknown mechanisms have been identified: RipAR, RipAW, RipG family, RipAB, RipA5, RipAD, RipAF1, RipD, RipE1, RipI, RipQ, RipAC, RipAP, RipAU, RipH1, RipM, RipS1, RipAN, and RipB. RSSC T3Es can also be perceived in planta by intracellular immune‐Nod‐like receptors (NLRs), leading to the activation of specific defence mechanisms, often associated with an HR. RipE1, RipAA, RipP1, RipX, RipP2, RipAT, RipAV, RipA1‐A5, RipTPS, RipAX2, RipAB, RipB, RipBN, and RipI also induce HR on several hosts. Some T3Es can modulate the activity of others and prevent their recognition by the plant surveillance system. Indeed, peroxisome‐localized RipAK suppresses effector‐triggered HR by inhibiting host catalase activities (CATs). RipAY and RipAC inhibit RipE1‐mediated HR

As model root and vascular plant pathogens, RSSC bacteria are among the pathogens with a larger number of functionally characterized T3Es. Some effectome‐scale experiments have tried to shed light on the function of RSSC T3Es through systematic determination of their ability to induce a hypersensitive response (HR; Wroblewski *et al*., 2009), inhibit plant defences (Nakano and Mukaihara, 2019a), or identify their plant targets (González‐Fuente *et al*., 2020). However, most of our current knowledge on effector function comes from smaller‐scale experiments in which often one or a few T3Es are studied. To date, we have counted more than 50 different RSSC T3Es that have been characterized with varying degrees of detail (Figure 1 and Table 1). One of the main factors complicating this task is the observed genetic redundancy among different RSSC T3Es (Angot *et al*., 2006; Solé *et al*., 2012; Chen *et al*., 2014). This redundancy is likely to ensure a more robust virulence strategy for the bacteria (Ghosh and O’Connor, 2017), although it makes the functional dissection of single effectors more complicated, particularly for the paralog families. Nevertheless, some members of these families can still have specific and nonredundant functions (Angot *et al*., 2006; Turner *et al*., 2009; Wang *et al*., 2016).

**TABLE 1:** List of functionally characterized *Ralstonia solanacearum* species complex type III effectors

Effector^a^	Functional annotation^b^	Homologs^c^	Subcellular localization	PAMP‐triggered immunity inhibition^d^	Description	Reference(s)
RipA (AWR) family			Cytoplasm (RipA1 and RipA4 also plasma membrane)	(+)	Collective contribution to virulence in eggplant and tomato and negative contribution to virulence in *Arabidopsis thaliana*	Cunnac *et al*. (2004b), Solé *et al*. (2012)
RipA1			Cytoplasm and plasma membrane		Cell death in *Nicotiana benthamiana*	Solé *et al*. (2012), Jeon *et al*. (2020)
RipA2			Cytoplasm		Major contribution to virulence in tomato, eggplant and *Arabidopsis* and cell death in different *Nicotiana* spp.	Cunnac *et al*. (2004b), Solé *et al*. (2012)
RipA4			Cytoplasm		Cell death in *Nicotiana glutinosa*	Solé *et al*. (2012)
RipA5			Cytoplasm	+	Inhibition of TOR pathway in yeast and in *N. benthamiana*, negative contribution to virulence in *A. thaliana* and cell death in different *Nicotiana* spp.	Solé *et al*. (2012), Popa *et al*. (2016), Nakano and Mukaihara (2019a)
RipAA (AvrA)					Cell death in pepper and different *Nicotiana* spp., contribution to virulence in *Medicago truncatula* and bacterial fitness in tomato	Carney and Denny (1990), Robertson *et al*. (2004), Pouemyro *et al*. (2009), Turner *et al*. (2009), Wroblewski *et al*. (2009), Macho *et al*. (2010), Chen *et al*. (2018)
RipAB	Nuclear localization signal		Nucleus	+	Contribution to virulence in potato and cell death in *N. benthamiana* only when localized in the nucleus	Zheng *et al*. (2019)
RipAC (PopC)	Leucine‐rich repeat domain	XopL/XopAE (X)	Nucleus and cytoplasm	+	Contribution to virulence in *A. thaliana* and tomato and bacterial fitness in eggplant and suppression of SGT1‐dependent immune response in *A. thaliana* and *N. benthamiana*	Macho *et al*. (2010), Nakano and Mukaihara (2019a), Yu *et al*. (2020)
RipAD		XopV (X)	Cytoplasm and chloroplasts	+	Inhibition of flg22‐induced reactive oxygen species production in *N. benthamiana*	Jeon *et al*. (2020)
RipAF1	Putative ADP‐ribosyltransferase	HopF2 (P)	Nucleus and cytoplasm	+	Contribution to bacterial fitness in eggplant and inhibition of flg22‐induced reactive oxygen species production in *N. benthamiana*	Macho *et al*. (2010), Jeon *et al*. (2020)
RipAK			Peroxisomes		Contribution to bacterial fitness in eggplant and inhibition of plant catalase activity to inhibit plant defence responses in *A. thaliana* and *Nicotiana tabacum*	Macho *et al*. (2010), Sun *et al*. (2017)
RipAL	Putative lipase domain	Lipase (X)	Chloroplasts	+	Induction of jasmonic acid production to inhibit salicylic acid signalling in *A. thaliana* and pepper	Nakano and Mukaihara (2018, 2019a)
RipAM				+	Contribution to virulence in potato	Zheng *et al*. (2019)
RipAN				+	Contribution to virulence in potato	Zheng *et al*. (2019), Nakano and Mukaihara (2019a)
RipAP	Ankyrin repeats			+	Inhibition of flg22‐induced reactive oxygen speciesproduction in *N. benthamiana*	Nakano and Mukaihara (2019a)
RipAR	Ubiquitin ligase domain		Cytoplasm	+	Inhibition of PAMP‐triggered immunity depending on its E3 ubiquitin ligase activity	Nakano *et al*. (2017)
RipAT					Hypersensitive response in lettuce and certain pepper and tomato cultivars	Wroblewski *et al*. (2009)
RipAU				+	Inhibition of flg22‐induced reactive oxygen species production in *N. benthamiana*	Nakano and Mukaihara (2019a)
RipAV		HopAV1 (P)			Contribution to bacterial fitness in eggplant and hypersensitive response in lettuce and certain pepper and tomato cultivars	Wroblewski *et al*. (2009), Macho *et al*. (2010)
RipAW	Ubiquitin ligase domain		Cytoplasm	+	Inhibition of PAMP‐triggered immunity depending on its E3 ubiquitin ligase activity	Nakano *et al*. (2017, 2019a)
RipAX2 (Rip36)	Zn‐binding motif	HopH1 (P), XopG (X)			Avirulence in wild and cultivated eggplant	Nahar *et al*. (2014), Morel *et al*. (2018)
RipAY	γ‐glutamyl cyclotransferases		Nucleus and cytoplasm	+	Contribution to bacterial fitness in eggplant, depletion of glutathione in yeast, eggplant and *A. thaliana*, inhibition of salicylic acid‐mediated defences in *A. thaliana* and *N. benthamiana* and suppression of RipE1‐mediated hypersensitive response in *N. benthamiana*	Macho *et al*. (2010), Fujiwara et al. (2016, 2020, 2016, 2020), Mukaihara *et al*. (2016), Sang et al. (2018, 2020, 2018, 2020)
RipB	Inosine‐uridine nucleoside N‐ribohydrolase	HopQ1 (P), XopQ (X)			Roq1‐mediated resistance	Nakano and Mukaihara (2019b)
RipBH		EspL2 (Sa), ShET2 (Y)			Contribution to virulence in potato	Zheng *et al*. (2019)
RipBN	Putative cysteine protease	AvrRpt2 (P)			Ptr1‐mediated resistance	Mazo‐molina *et al*. (2019)
RipD		HopD1 (P), XopB (X)	Endoplasmic reticulum	+	Contribution to bacterial fitness in eggplant, tomato, and bean and inhibition of flg22‐induced reactive oxygen speciesproduction in *N. benthamiana*	Macho *et al*. (2010), Jeon *et al*. (2020)
RipE1	Transglutaminase protein family	HopX1 (P), XopE (X)		+	Induction of salicylic acid and jasmonic acid synthesis to trigger immunity in *N. benthamiana* and *A. thaliana*	Nakano and Mukaihara (2019a), Sang *et al*. (2020)
RipF1 (PopF1)	Translocator of T3E	NopX (B/Si)			Important for the translocation of effector	Meyer *et al*. (2006)
RipF2 (PopF2)	Translocator of T3E	NodX (B/Si)			Important for the translocation of effector	Meyer *et al*. (2006)
RipG (GALA) family	F‐box			(+)	Collective contribution to virulence in *A. thaliana*, tomato, and eggplant, interaction with SKP1‐like proteins (except RipG3 and RipG4)	Angot *et al*. (2006), Remigi *et al*. (2011), Wang *et al*. (2016)
RipG1	F‐box and N‐myristoylation domains		Chloroplasts and plasma membrane	+	Inhibition of flg22‐induced salicylic acid‐dependent defence responses in *N. benthamiana* and *A. thaliana*	Medina‐Puche *et al*. (2019)
RipG3	F‐box and N‐myristoylation domains		Chloroplasts	+	Inhibition of flg22‐induced salicylic acid‐dependent defence responses in *N. benthamiana* and *A. thaliana*	Medina‐Puche *et al*. (2019)
RipG4	F‐Box			+	Inhibition of callose deposition in *A. thaliana*	Remigi *et al*. (2011)
RipG7	F‐Box				Essential for virulence in late stages of infection in *M. truncatula*	Angot *et al*. (2006), Turner *et al*. (2009), Wang *et al*. (2016)
					Interaction with *A. thaliana* ASK1, 2, 11, and 13, and *M. truncatula* MSKa	
RipH (HLK) family		XopP (X)		(+)	Collective contribution to virulence in tomato	Chen *et al*. (2014), Nakano and Mukaihara (2019a)
RipI			Nucleus	+	Cell death in yeast and *N. benthamiana*, through interaction with bHLH93 transcription factor, and immune responses in tomato	Deng *et al*. (2016), Nakano and Mukaihara (2019a), Zhuo (2020)
RipM				+	Inhibition of flg22‐induced reactive oxygen speciesproduction in *N. benthamiana*	Nakano and Mukaihara (2019a)
RipN	Nudix hydrolase domain		Nucleus and endoplasmic reticulum	+	Alteration of the plant NADH/NAD^+^ ratio and suppression of PAMP‐triggered immunity defences in *A. thaliana*	Sun *et al*. (2019)
RipP1 (PopP1)	Putative acetyltransferase	HopZ2 (P), XopJ4 (X)			Avirulence factor in different *Nicotiana* spp. (major contribution in *N. glutinosa*) and in *Petunia* lines	Lavie *et al*. (2002), Pouemyro *et al*. (2009), Chen *et al*. (2018)
RipP2 (PopP2)	Acetyltransferase	AvrA (Sa), HopZ4 (P), VopA (V), YopJ (Y)	Nucleus	+	Acetylation of WRKY transcription factors to inhibit PAMP‐triggered immunity defences and RRS1‐R to induce effector‐triggered immunity in *A. thaliana*, avirulence factor in eggplant and contribution to virulence in *A. thaliana* and to bacterial fitness in tomato, eggplant, and bean	Deslandes *et al*. (2003), Tasset *et al*. (2010), Macho *et al*. (2010), Le Roux *et al*. (2015), Sarris *et al*. (2015), Xiao *et al*. (2015)
RipQ		HopAA1 (P)		+	Inhibition of flg22‐induced reactive oxygen speciesproduction in *N. benthamiana*	Nakano and Mukaihara (2019a)
RipR (PopS)		AvrE/HopR1 (P), DspA/E (E), XopAM (X)			Inhibition of salicyclic acid‐dependent defences and contribution to virulence in *Solanum* spp. and to bacterial fitness in eggplant	Macho *et al*. (2010), Jacobs *et al*. (2013)
RipS1 (SKWP1)		XopAD (X)		+	Inhibition of flg22‐induced reactive oxygen species production in *N. benthamiana*	Nakano and Mukaihara (2019a)
RipS4 (SKWP4)		XopAD (X)			Contribution to bacterial fitness in eggplant	Macho *et al*. (2010)
RipTAL (Brg11)	Transcription activator‐like protein	AvrBs3/TAL family (X)	Nucleus		Specific binding on DNA from different hosts and induction of synthesis of polyamines in *Solanum* spp., possibly to inhibit the proliferation of competitors, and contribution to bacterial fitness in eggplant	Macho *et al*. (2010), de Lange *et al*. (2013), Wu *et al*. (2019)
RipTPS	Trehalose‐6‐phosphate‐synthase	Trehalose‐6‐phosphate synthase (A/X)			Synthesis of trehalose‐6‐phosphate in yeast and enzymatic activity‐independent hypersensitive response in *N. tabacum*	Pouemyro *et al*. (2014)
RipX (PopA)	Hairpin‐like protein		Nucleus and plasma membrane		Hypersensitive response in *Petunia*, *N. tabacum* and *N. benthamiana* by affecting negatively the transcription of *atpA* gene for the latter and formation of ion‐conducting pores in vitro	Arlat *et al*. (1994), Belbahri *et al*. (2002), Racapé *et al*. (2005), Sun *et al*. (2020)
RipY					Contribution to bacterial fitness in eggplant	Macho *et al*. (2010)

^a^ Former name in parentheses.

^b^ Proven or putative functional annotation.

^c^ Homologs characterized in other bacterial genera. A, *Acidovorax*; B, *Bradyrhizobium*; E, *Erwinia*; P, *Pseudomonas*; Sa, *Salmonella*; Si, *Sinorhizobium*; V, *Vibrio*; X, *Xanthomonas*; Y, *Yersinia*.

^d^ Indicated only when the ability to inhibit any classical PAMP‐triggered immunity (PTI) response has been proven. In parentheses when only some members of a paralog T3E family members inhibit PTI responses.

Similar to other pathogens, RSSC T3Es collectively contribute to the pathogen fitness in the plant through different and not always well‐characterized mechanisms (Toruño *et al*., 2016). These include the interference with the plant basal defence responses, alteration of the plant metabolism, and avoidance of the specific recognition of other T3Es. However, some RSSC T3Es can also be recognized by specific plant genotypes and induce strong immune responses.

### 3.1 Interference with plant basal immunity

The subversion of basal defences is one of the most studied functions of pathogen effectors. Several RSSC T3Es are known to interfere with different host cellular processes involved in these basal defence responses. RipP2 (former PopP2) relies on its acetyltransferase activity to acetylate the WRKY domain of the plant homonymous transcription factors, which prevents their association with DNA and subsequent expression of defence‐related genes (Le Roux *et al*., 2015). RipAY is selectively activated by eukaryotic thioredoxins to degrade the host glutathione, which plays an important role in plant immunity (Fujiwara *et al*., 2016 , 2020; Mukaihara *et al*., 2016; Sang *et al*., 2018). RipAR and RipAW rely on their E3 ubiquitin ligase activity to inhibit plant defence responses (Nakano *et al*., 2017). Also linked to ubiquitination, the RipG (former GALA) family of T3Es presents a eukaryotic F‐box domain required for the interaction with *Arabidopsis* components of the Skp, Cullin, F‐box containing (SCF) complex contributing to *Ralstonia* virulence (Angot *et al*., 2006; Remigi *et al*., 2011). RipAL is a chloroplastic effector with a lipase domain required for the induction of jasmonic acid (JA) production and suppression of salicylic acid (SA) signalling (Nakano and Mukaihara, 2018). The inhibition of SA‐mediated defences seems also to be the role of RipR (former PopS) and RipG1 and RipG3, although the molecular mechanisms behind this inhibition still remain unknown (Jacobs *et al*., 2013; Medina‐Puche *et al*., 2019). RipAB (former PopB) down‐regulates the calcium signalling pathway and inhibits the plant basal defences (Zheng *et al*., 2019). Finally, RipN contains a Nudix hydrolase domain required to alter the NADH/NAD^+^ ratio in planta and to inhibit the plant defence responses (Sun *et al*., 2019).

In addition to these functionally characterized RSSC T3Es, other basal defence inhibiting T3Es have been identified in large‐scale screenings. Sixteen additional RSSC T3Es have been reported as suppressors of the flg22‐induced reactive oxygen species (ROS) production, a marker typically associated with pathogen‐associated molecular pattern (PAMP)‐triggered immunity (Sang and Macho, 2017): RipA5 (former AWR5), RipAD, RipAF1, RipD, RipE1, RipI, RipQ, RipAC (former PopC), RipAL, RipAP, and RipAU; and to a lesser extent RipH1 (former HLK1), RipM, RipS1 (former SKWP1), RipAN, and RipB (Nakano and Mukaihara, 2019a; Jeon *et al*., 2020).

### 3.2 Targeting plant metabolism

Plant pathogenic bacterial T3Es can also interfere with different host metabolic processes to promote the bacterial survival, release nutrients, and facilitate the infection (Macho, 2016). RSSC bacteria thrive in the xylem, manipulating the composition of the xylem sap (Lowe‐Power *et al*., 2018). This manipulation can occur through different mechanisms, including the T3SS, as RSSC bacteria are able to inject T3Es into living cells surrounding the vasculature (Vasse *et al*., 2000; Henry *et al*., 2017). Indeed, some RSSC T3Es display different activities that could modulate the plant metabolism. One of the better characterized examples is RipTAL (former Brg11), which presents homology with *Xanthomonas* spp. transcription activator‐like (TAL) effectors (de Lange *et al*., 2013). RipTAL induces the expression of plant genes involved in the synthesis of polyamines, evading their native translational regulation mechanisms (Wu *et al*., 2019). It is hypothesized that this RipTAL‐induced boost of the plant polyamine levels prevents the proliferation of possible *Ralstonia* competitors (Wu *et al*., 2019). RipA5 acts as an inhibitor of the conserved target of rapamycin (TOR) pathway in yeast and plant cells (Popa *et al*., 2016). As a key regulator of the switch between growth and stress responses (Dobrenel *et al*., 2016), RipA5‐mediated inhibition of the plant TOR pathway leads to reduced nitrate reductase activity (Popa *et al*., 2016). Lastly, RipTPS possesses trehalose‐6‐phosphate synthase activity in yeast (Poueymiro *et al*., 2014). As trehalose‐6‐phosphate is a key regulatory molecule in plant metabolism (Baena‐González and Lunn, 2020), RipTPS could potentially interfere with this regulation but so far this activity has not been shown in planta.

### 3.3 Contribution to virulence through (as of yet) unknown mechanisms

In addition to the beforementioned RSSC T3Es for which functional roles could be assigned, other *T3E* genes have been also identified as contributors to bacterial virulence on different hosts. These additional *T3E* genes have been identified through pathogenicity or competitive index assays with single or multiple gene mutants. These tests allow us to pinpoint the involvement in virulence but do not provide further information about the underlying molecular mechanisms. This is the case for RipA2 and RipD, which contribute to virulence in tomato (Cunnac *et al*., 2004b), or RipAA and RipG7, important in the early and late stages of infection of the model legume species *Medicago truncatula*, respectively (Turner *et al*., 2009; Wang *et al*., 2016). RipAC, RipAF1, RipAK, RipAV, RipAY, RipD, RipP2, RipR, RipS4, RipY, and RipTAL contribute to bacterial fitness in eggplant (Macho *et al*., 2010). For RipD and RipP2, this contribution to fitness was also demonstrated in tomato and bean, and in the case of RipAA, exclusively in tomato (Macho *et al*., 2010). The RipA family members contribute collectively to virulence in both eggplant and tomato (Solé *et al*., 2012), and the RipH family members also contribute to virulence in tomato (Chen *et al*., 2014). RipAM, RipAN, and RipBH contribute significantly to virulence in potato (Zheng *et al*., 2019), and RipAC acts similarly in tomato (Yu *et al*., 2020).

### 3.4 Effectors triggering plant immune responses

Through evolution, plants have evolved mechanisms to recognize specific RSSC T3Es and induce a strong defence response often associated with a hypersensitive response (HR) (Balint‐Kurti, 2019). This is precisely what was observed on petunia with RipX (former PopA), the first RSSC T3E to have been characterized (Arlat *et al*., 1994). This same phenotype was later observed in tobacco (Belbahri *et al*., 2002; Racapé *et al*., 2005), and could be explained by a RipX‐mediated inhibition of the gene expression of the ATP synthase F1 subunit α (Sun *et al*., 2020). RipAA and RipP1 (former AvrA and PopP1, respectively) trigger strong HRs in diverse *Nicotiana* spp. (Carney and Denny, 1990; Robertson *et al*., 2004; Poueymiro *et al*., 2009; Chen *et al*., 2018). Additionally, RipP1 also triggers anHR on petunia St40 line (Lavie *et al*., 2002), and RipAA, in pepper CW300 and RNaKy accessions (Wroblewski *et al*., 2009). RipP2 was the first RSSC T3E for which the corresponding immune receptor was identified in *Arabidopsis*: Recognition of *R. solanacearum* 1 (RRS1) (Deslandes *et al*., 1998, Deslandes *et al*., 2003). It was later shown that this recognition also involves the Resistance to *Pseudomonas syringae* 4 (RPS4) immune receptor (Gassmann *et al*., 1999; Narusaka *et al*., 2009; Williams *et al*., 2014). The RPS4/RRS1‐dependent immunity is activated by RipP2 acetylation of RRS1 C‐terminal WRKY domain representing an integrated decoy that mimics RipP2 virulence targets (Tasset *et al*., 2010; Le Roux *et al*., 2015; Sarris *et al*., 2015). RipAT and RipAV induce HR‐like phenotypes when expressed in most lettuce and certain pepper and tomato cultivars (Wroblewski *et al*., 2009). RipA1, RipA2, RipA3, and RipA5 trigger HRs with varying intensities on different *Nicotiana* spp. (Solé *et al*., 2012; Jeon *et al*., 2020). RipTPS produces an HR specifically on *N. tabacum* independently of its enzymatic activity (Poueymiro *et al*., 2014). RipAX2 (former Rip36) elicits immunity on wild and cultivated eggplants in a Zn‐finger domain‐dependent (Nahar *et al*., 2014) and independent (Morel *et al*., 2018) manner, respectively. RipAB triggers an HR in *N. benthamiana* but only when localized in the nucleus (Zheng *et al*., 2019). RipB induces chlorosis in different *Nicotiana* spp. in a Recognition of XopQ1 (Roq1)‐dependent manner (Nakano and Mukaihara, 2019b). RipBN triggers resistance in tomato in a *Pseudomonas tomato race 1* (*Ptr1*)‐dependent manner (Mazo‐Molina *et al*., 2019). RipE1 triggers immune responses mediated by both SA and JA in *N. benthamiana* and *Arabidopsis* (Sang *et al*., 2020). RipE1 also triggers an HR in *N. tabacum* and *N. benthamiana* in a Suppressor of G2 allele of *skp1* (SGT1)‐dependent manner for the latter (Jeon *et al*., 2020). Last, RipI triggers immune responses in tomato and cell death in yeast and *N. benthamiana*, the latter through interaction with the plant basic helix‐loop‐helix 93 (bHLH93) transcription factor (Deng *et al*., 2016; Zhuo *et al*., 2020).

### 3.5 Effectors preventing other effectors to be recognized in planta

The recognition of RSSC T3Es and subsequent strong immune responses can also be counteracted through the action of other T3Es, sometimes referred as “meta‐effectors” (Kubori *et al*., 2010). This could allow the bacteria to conserve effectors with potent virulence functions for which a given host has already developed specific recognition capabilities. This is the case for RipAY, which can inhibit the previously mentioned RipE1‐triggered immunity (Sang *et al*., 2020). RipAY inhibits RipE1‐mediated activation of the SA signalling pathway probably through degradation of the plant cellular glutathione (Mukaihara *et al*., 2016; Sang *et al*., 2018 , 2020). It has also been proposed that RipAC suppresses RipE1‐triggered immunity, inhibiting in this case SGT1‐mediated MAPK activation (Yu *et al*., 2020). RipAK is able to prevent *Ralstonia*‐induced HR in *N. tabacum* by inhibiting plant catalase activity (Sun *et al*., 2017). Whether this HR is induced by RipAA, RipB, and/or RipP1, responsible for RSSC incompatibility in *N. tabacum* (Poueymiro *et al*., 2009; Nakano and Mukaihara, 2019b), is still unknown.

## 4 CONCLUSIONS AND PERSPECTIVES

In this microreview, we have summarized the current knowledge about RSSC T3Es. Despite being one of the largest and most studied bacterial plant pathogen effectomes, a majority of RSSC T3Es remain poorly characterized to date. This will undoubtedly change in the near future as more and more RSSC T3Es are currently being characterized by several research groups worldwide. Nevertheless, from what is currently known, we can already see that the large RSSC effectome is highly diversified in terms of molecular functions, subcellular localizations, and host‐targeted processes. RSSC T3Es act in the host plasma membrane, cytoplasm, nucleus, chloroplasts, or peroxisomes, and interfere with the plant gene expression regulation at the transcriptional and translational level, metabolism, ubiquitination, phytohormone production and signalling, redox homeostasis, and calcium signalling. This functional repertoire, coupled with genetic and functional redundancy, confers RSSC bacteria with a strong, varied, and robust set of weaponry against their hosts. It is thus tempting to hypothesize that this T3E diversity contributes to the adaptability of *Ralstonia* as a species complex to a wide range of plant hosts. It should also be noted that this large cornucopia of T3Es could be a key factor in the appearance of RSSC strains adapted to new host plants, like the recently identified strains virulent on cucurbitaceous crops (Wicker *et al*., 2007), coffee plant (Lopes *et al*., 2015), fig tree (Jiang *et al*., 2016), African daisy (Weibel *et al*., 2016), and roses (Tjou‐Tam‐Sin *et al*., 2017). Future work will help to elucidate whether the so far uncharacterized T3Es target similar processes to those previously described or if, on the contrary, they interfere with completely different plant processes. This is key to understanding whether the strength of RSSC effectomes comes from its high diversity (i.e., RSSC bacteria target simultaneously many different plant processes) or from its redundancy (i.e., RSSC bacteria target a few key plant processes with redundant T3Es). The characterization of new T3Es will also allow the plant processes that RSSC bacteria specifically target to be determined to establish a successful infection. Interestingly, 9 out the 16 RSSC core T3Es have been shown to contribute to virulence in different hosts: RipA2, RipAB, RipAM, RipAN, RipAY, RipG5, RipG6, RipH2, and RipR. From these nine T3Es, functional information is only available for five of them: RipG5 and RipG6 interact with components of the E3 ubiquitin ligase complex (Angot *et al*., 2006; Remigi *et al*., 2011), RipR inhibits SA‐mediated defence responses (Jacobs *et al*., 2013), RipAY degrades plant glutathione (Fujiwara *et al*., 2016 , 2020; Mukaihara *et al*., 2016; Sang *et al*., 2018), and RipAB down‐regulates the calcium signalling pathway (Zheng *et al*., 2019). These different processes, together with the unknown ones targeted by the other core T3Es, could represent the minimum plant processes that *Ralstonia* needs to modulate. This “basal arsenal” could be complemented with accessory T3Es that could have additive effects, targeting the same or different processes. However, this characterization might prove quite complex as these plant processes, and their modulation by *Ralstonia* T3Es, might vary substantially among different organs and host species. The diverse, and sometimes large, host range of RSSC strains and the functional diversity and redundancy of its effectome are therefore some of the causes of RSSC adaptability and aggressiveness, but also some of the major factors complicating its systematic and exhaustive study. A valuable tool that will open a wide variety of possibilities in the decipherment of RSSC T3E functions is the generation of a strain devoid of all its effectors, as has been performed on the *P. syringae* strain DC3000 (Cunnac *et al*., 2011). This should be completed soon on the RSSC strain OE1‐1 (K. Onishi, Kochi University, Japan, personal communication). The fact that RSSC bacteria can infect both model and agronomically important crop species confers a practical perspective to this information gathered over the last decades. This should certainly contribute to the design of effective and sustainable control measures against the devastating RSSC.

## Data Availability

Data sharing is not applicable to this article as no new data were created or analysed in this study.
